# Increased Platelet Reactivity and Proinflammatory Profile Are Associated with Intima–Media Thickness and Arterial Stiffness in Prediabetes

**DOI:** 10.3390/jcm11102870

**Published:** 2022-05-19

**Authors:** Maurizio Di Marco, Francesca Urbano, Agnese Filippello, Stefania Di Mauro, Alessandra Scamporrino, Nicoletta Miano, Giuseppe Coppolino, Giuseppe L’Episcopo, Stefano Leggio, Roberto Scicali, Salvatore Piro, Francesco Purrello, Antonino Di Pino

**Affiliations:** Department of Clinical and Experimental Medicine, University of Catania, 95131 Catania, Italy; maurizio.dimarco@studium.unict.it (M.D.M.); francescaurbano@hotmail.it (F.U.); agnese.filippello@gmail.com (A.F.); 8stefaniadimauro6@gmail.com (S.D.M.); alessandraska@hotmail.com (A.S.); nicoletta.miano@gmail.com (N.M.); giuseppecoppolino93@gmail.com (G.C.); peppe94@gmail.com (G.L.); stefano_leggio@yahoo.it (S.L.); robertoscicali@gmail.com (R.S.); salvatore.piro@unict.it (S.P.); nino_dipino@hotmail.com (A.D.P.)

**Keywords:** prediabetes, 11-dh-thromboxane, cardiovascular risk, IMT, arterial stiffness

## Abstract

Alterations of glucose homeostasis are associated with subclinical vascular damage; however, the role of platelet reactivity in this process has not been fully investigated. In this cross-sectional study, we evaluated the correlation between markers of platelet reactivity and inflammation and markers of vascular disease in subjects with prediabetes. Markers of platelet reactivity such as 11-dehydro-thromboxane B2 urinary levels (11-dh-TXB2) and mean platelet volume (MPV) and inflammatory indexes such as platelet-to-lymphocyte ratio (PLR) were evaluated in subjects with prediabetes (*n* = 48), new-onset type 2 diabetes (NODM, *n* = 60) and controls (*n* = 62). Furthermore, we assessed the cardiovascular risk profile of the study population with arterial stiffness and quality intima–media thickness (qIMT). Subjects with prediabetes and NODM exhibited higher 11-dh-TXB2 urinary levels and MPV and a proinflammatory profile with an increased PLR, high-sensitivity C-reactive protein, ferritin and fibrinogen. Furthermore, after multiple regression analyses, we found that urinary 11-dh-TXB2 was one of the major determinants of IMT and arterial stiffness parameters. In conclusion, subjects with prediabetes exhibit increased platelet reactivity as well as a proinflammatory profile. Furthermore, this condition is associated with early markers of cardiovascular disease.

## 1. Introduction

Prediabetes identifies a clinical condition with a higher risk of developing diabetes and cardiovascular disease [1]. According to the American Diabetes Association (ADA), this category includes subjects with impaired fasting glucose (IFG) and/or impaired glucose tolerance (IGT) and/or HbA1c 5.7–6.4% [2].

It is well known that mechanisms at the base of macrovascular complications of diabetes already act in the prediabetes phase, prior to diagnosis of diabetes, via atherosclerosis [3,4,5]. Based on these considerations, prediabetes is associated with more advanced vascular damage compared with normoglycemia [6]. In the last few years, several different pathways have been analyzed to explain the link between early alterations of glucose homeostasis and vascular damage, such as the role of advanced glycation end-products and their soluble receptors [7], the increase in small, dense low-density lipoproteins (sdLDLs) [8], low vitamin D plasma levels [9] and micro-RNA (mi-RNA) deregulation [10].

Even though platelets play a pivotal role in the pathogenesis of atherothrombosis, the role of platelet reactivity in this process has not been fully investigated, especially in the setting of prediabetes [11]. This phenomenon has been studied in other conditions related to metabolic syndrome, such as overweight, obesity and insulin resistance [12,13,14].

Platelet activation is quite a complex mechanism, and platelet reactivity can be evaluated both with morphological and functional parameters, i.e., mean platelet volume (MPV) and 11-dehydro-thromboxane B2 (11-dh-TXB2) urinary excretion. In physiological conditions, in response to external stimuli, platelets increase their thromboxane A2 (TXA2) production, while prostacyclin production is reduced. This phenomenon is regulated by the activity of cyclooxygenases (COXs) [15]. The importance of platelet morphological features can be explained considering that larger platelets are more active because of the presence of a higher quantity of alpha-granules and the production of more thromboxane A2 (TXA2) [16]. TXA2 is chemically instable, and thus it is rapidly converted into thromboxane B2 (TXB2) that, in turn, is metabolized into 11-dh-TXB2. 11-dh-TXB2 is the principal metabolite of TXA2, and different studies have shown the possibility of using its urinary excretion as a biomarker of platelet reactivity [17,18].

Platelets are essential for primary hemostasis and repair of the endothelium; however, they also play a key role in the development of vascular inflammation and participate in the process of forming and extending atherosclerotic plaques. The relationship between chronic and acute vascular inflammation is unclear, but platelets are a source of inflammatory mediators; i.e., the F2 isoprostane 8-iso-PGF2α can be produced as a minor product of the cyclooxygenase activity of platelets in response to platelet stimulation with collagen, thrombin or arachidonate, and it is also a marker of oxidative stress [18,19]. In addition, it is interesting to consider the platelet-to-lymphocyte ratio (PLR), which is a cheap and easy way to obtain a parameter that takes into account two different aspects of atherosclerosis: platelet count and inflammation. This index has been used in the field of oncology, but in the last few years, it has become a promising biomarker in other conditions, particularly in cardiovascular disease [20,21,22]. In the atherosclerotic process, the inflammatory response and oxidative stress also play important roles, and the activation of platelets by inflammatory triggers may be a critical component of vascular damage [23,24].

In this study, we evaluated platelet reactivity and inflammatory parameters in subjects with prediabetes and new-onset type 2 diabetes (NODM) and examined their association with early markers of cardiovascular disease.

## 2. Materials and Methods

*Study subjects*. One hundred seventy subjects with no previous diagnosis of diabetes who attended our university hospital for diabetes and cardiovascular risk evaluation were consecutively recruited in this study. The inclusion criteria were ages ranging from 18–65 years and Caucasian race. All patients underwent a physical examination and review of clinical history, smoking status (active or nonsmokers) and alcohol consumption. The exclusion criteria were as follows: a previous history of diabetes; a previous history of overt cardiovascular events (stroke, ischemic heart disease, chronic obstructive peripheral arteriopathy or heart failure), anemia or hemoglobinopathies; use of medications known to affect glucose metabolism and platelet aggregation (statins, antiplatelet drugs); clinical evidence of advanced liver or renal disease, chronic inflammatory disease or other chronic diseases; and/or recent history of acute illness, malignant disease and drug or alcohol abuse.

BMI was calculated as weight (kg)/(height (m))^2^. Blood pressure (BP) was measured with a calibrated sphygmomanometer after 10 min rest. Venous blood samples were drawn from the antecubital vein in the morning after an overnight fast. Baseline venous blood samples were obtained for the measurement of clinical biochemistry parameters. LDL cholesterol concentrations were estimated using the Friedewald formula. All subjects underwent a 75 g oral glucose tolerance test (OGTT) with 0, 30, 60, 90, and 120 min sampling for plasma and insulin, as previously described [25]. Glucose tolerance status was defined on the basis of OGTT according to ADA recommendations [2].

*Biochemical analyses*. Plasma glucose, serum total cholesterol, triglycerides, high-density lipoprotein (HDL) cholesterol and high-sensitivity C-reactive protein (hs-CRP) were measured using available enzymatic methods, as previously described [26].

The concentrations of urinary 11-dh-TXB2 and 8-iso-PGF2α were measured with an enzyme-linked immunosorbent assay commercial kit (Cayman Chemical, Ann Arbor, MI, USA). Data are expressed as ng/mg creatinine. Analyses were performed in a blinded manner. Thus, the biologist who analyzed the samples was not aware of the clinical characteristics of the subjects. The commercially available ELISA kits were used according to the manufacturer’s instructions.

HbA1c was measured via high-performance liquid chromatography using a National Glycohemoglobin Standardization Program and was standardized to the Diabetes Control and Complications Trial (DCCT) assay reference [27]. Chromatography was performed using a certified automated analyzer (HPLC; HLC-723G7 hemoglobin HPLC analyzer; Tosoh Corp.) (normal range 4.25–5.9%).

*Carotid ultrasound examination*. Ultrasound scans were performed using a high-resolution B-mode ultrasound system equipped with a linear array transducer. All ultrasound examinations were performed by a single physician who was blinded to the clinical and laboratory characteristics of the subjects. Longitudinal B-mode (60 Hz, 128 radiofrequency lines) images of the right common carotid artery 2 cm below the carotid bulb were obtained using a high-precision echo tracking device (MyLab Alpha, Esaote, Maastricht, The Netherlands) paired with a high-resolution linear array transducer (13 MHz) to acquire quality intima–media thickness (qIMT) using the built-in echo tracking software.

*Pulse wave velocity*. The SphygmoCor CvMS (AtCor Medical, Sydney, Australia) system was used for the determination of the pulse wave velocity (PWV), as previously described [28]. This system uses a tonometer, and two different pressure waves obtained at the common carotid artery (proximal recording site) and at the femoral artery (distal recording site). An electrocardiogram was used to determine the start of the pulse wave. The PWV was determined as the difference in travel time of the pulse wave between the two different recording sites and the heart, divided by the travel distance of the pulse waveform. The PWV was calculated on the mean basis of 10 consecutive pressure waveforms to cover a complete respiratory cycle.

*Pulse wave analysis*. All measurements were made from the right radial artery by applanation tonometry using a Millar tonometer (SPC-301; Millar Instruments, Houston, TX, USA) [29]. The measurements were performed by a single investigator with the subject in the supine position. The data were collected directly with a desktop computer and processed with SphygmoCorCvMS (AtCor Medical, Sydney, Australia). The aortic waveform has two systolic pressure peaks; the latter one is caused by wave reflection from the periphery. With arterial stiffening, both the PWV and the amplitude of the reflected wave are increased such that the reflected wave arrives earlier and adds to (or augments) the central systolic pressure. The aortic waveform in pulse wave analysis was subjected to further analysis for the calculation of the aortic augmentation pressure (AugP), augmentation index (AugI—calculated by dividing augmentation by pulse pressure), central BP, ejection duration (duration of the systolic period in milliseconds) and Buckberg subendocardial viability ratio (SEVR; area of diastole divided by area of systole during one cardiac cycle in the aorta). Pulse pressure is the difference between the systolic and diastolic BPs.

*Statistical analyses*. The sample size was calculated based on 11-dh-TXB2 using a level of significance (α) set to 5% and a power (1 − β) set to 80%. The estimated sample size was 48 subjects per group.

Statistical comparisons of clinical and biomedical parameters were performed using Stat View 6.0 for Windows. The data are presented as the mean ± standard deviation (SD) or median and interquartile range (IQR). Each variable’s distributional characteristics, including normality, were assessed by the Kolmogorov–Smirnov test. ANOVA for clinical and biological data was performed to test the differences among groups, and the Bonferroni post hoc test for multiple comparisons was further performed. The χ2 test was used for categorical variables. A *p* value less than 0.05 was considered significant. When necessary, numerical variables were logarithmically transformed to reduce skewness.

Simple regression analysis was performed to relate 11-dh-TXB2, MPV and PLR to the following variables: age, sex, BMI, systolic and diastolic BP, HDL cholesterol, triglycerides, LDL cholesterol, homeostasis model assessment insulin resistance index (HOMA-IR), HbA1c and fasting glucose.

In order to identify variables independently associated with variations in qIMT, PWV and AugP, we performed two multivariate regression models: the first model included cardiovascular risk factors (age, sex, BMI, systolic and diastolic BP, LDL cholesterol, HDL cholesterol, HbA1c, fasting glucose, HOMA-IR); variables reaching significance in the first model were included in a second model including variables related to platelet activation and inflammation (11-dh-TXB2, platelet count, MPV, 8-iso-PGF2α, PLR, hs-CRP, fibrinogen).

The variance inflation factor (VIF) was used to check for the problem of multicollinearity among the predictor variables in multiple regression analysis. Any variable with a VIF that exceeded 4 was excluded from the model, as recommended in the literature (no variable was detected with a VIF greater than 4) [30].

The study was approved by the local ethics committee. Informed consent was obtained from each participant.

## 3. Results

The study population (170 subjects) was divided into three groups based on fasting glucose, OGTT and HbA1c levels: 62 control subjects (36%) (control group) (NFG and NT and HbA1c < 5.7%), 48 subjects with prediabetes (28%) (prediabetes group) (IFG and/or impaired glucose tolerance (IGT) and/or HbA1c 5.7–6.4%) and 60 subjects with NODM (35%) (NODM group) (fasting glucose ≥ 126 mg/dL and/or 2 h glucose post-OGTT ≥ 200 mg/dL and/or HbA1c ≥ 6.5%). The clinical and biochemical characteristics of the study subjects are presented in Table 1. The prediabetic patients were not older than the controls and had a similar BMI. Subjects with prediabetes were younger than subjects with NODM; however, there were no differences concerning BMI and diastolic BP. In addition, subjects with NODM showed a higher HOMA index than those of the prediabetic and control groups.

### 3.1. Platelet Reactivity and Inflammation Indexes in Subjects with Prediabetes

MPV was in prediabetic subjects significantly higher than that in controls (9.1 ± 0.8 vs. 8.7 ± 1.1 fL, *p* < 0.05) and showed no significant differences from that in subjects with NODM. No differences were found in 8-iso-PGF2α urinary levels between the three groups.

The platelet count in the subjects with prediabetes was higher than that in controls without reaching statistical significance (230 ± 64.2 vs. 251.5 ± 69 103/µL, *p* = 0.15) but was similar to that in subjects with NODM.

In the simple regression analysis, 11-dh-TXB2 urinary levels were associated with age (r = 0.197, *p* = 0.01) and systolic blood pressure (r = 0.14, *p* < 0.05). Moreover, MPV was associated with fasting glucose (r = 0.25, *p* = 0.001), HbA1c (r = 0.16, *p* < 0.05) and HOMA-IR (r = 0.25, *p* = 0.0018).

Hs-CRP was significantly higher in prediabetic subjects in comparison with controls (0.21 (0, 12–0.54) vs. 0.09 (0.07–0.29) mg/dL, *p* < 0.05) and in diabetic subjects than in controls (0.28 (0.19–0.59) vs. 0.09 (0.07–0.29) mg/dL, *p* < 0.05). Ferritin was significantly higher in the prediabetes group in comparison with the control group (119 (73–120) vs. 68 (73–200) ng/mL, *p* < 0.05) and in the NODM group than in the control group (108 (40.5–192.5) vs. 68 (73–200) ng/mL, *p* < 0.05). Fibrinogen in the prediabetes group was significantly higher than that in the control group (350 ± 71.3 vs. 324 ± 73.1 mg/dL, *p* < 0.05) and showed no significant differences from that in the NODM group. PLR was higher in the prediabetes group than in the control group without reaching statistical significance (138 ± 54 vs. 121.2 ± 38, *p* = 0.16), and it was significantly higher in the NODM group than in the control group (144 ± 87 vs. 121.2 ± 38, *p* < 0.05) (Table 2).

PLR was associated with diastolic BP (r = 0.28, *p* = 0.0002), HbA1c (r = 0.16, *p* = 0.04) and HOMA-IR (r = 0.15, *p* < 0.05) in the simple regression analysis.

### 3.2. Intima–Media Thickness and Arterial Stiffness in Subjects with Prediabetes

We found several differences in intima–media thickness and arterial stiffness parameters between the study groups (Table 3).

qIMT in the prediabetes group was significantly higher than that in the control group (0.75 ± 0.12 vs. 0.69 ± 0.11 mm, *p* < 0.05) and had no differences from that in the NODM group.

In the multiple regression analysis using two models (see Section 2), qIMT exhibited a significant correlation with age (*p* < 0.0001) and fasting glucose (*p* = 0.01) in the first model. In the second model, the variables that remained significantly associated with qIMT were age (*p* < 0.0001), 11-dh-TXB2 urinary levels (*p* < 0.05), 8-iso-PGF2α urinary levels (*p* = 0.04) and fibrinogen (*p* < 0.05) (Table 4).

PWV was significantly higher in the prediabetes group vs. the control group (7.9 ± 1.5 vs. 7.4 ± 1 m/s, *p* < 0.05) and in NODM vs. the prediabetes group and the control group (8.1 ± 1.9 vs. 7.9 ± 1.5 and 7.4 ± 1 m/s, *p* < 0.05). AugP was significantly higher in the prediabetes group than in the control group (12 ± 8 vs. 10 ± 4.8 mmHg, *p* < 0.05) and in NODM in comparison with the control group (12.7 ± 5.5 vs. 10 ± 4.8 mmHg, *p* < 0.05). AugI was higher in the prediabetes group than in the control group without reaching statistical significance (28.8 ± 13.1 vs. 26 ± 8.2%, *p* = 0.08), while it was significantly higher in the NODM group (30.5 ± 9.7 vs. 26 ± 8.2%, *p* < 0.05).

As regards the multiple regression analysis, in the first model, PWV showed a statistically significant association with male sex (*p* = 0.03), systolic BP (*p* < 0.0001) and HDL cholesterol (*p* = 0.01), while in the second model the variables that remained significantly related to PWV were male sex (*p* = 0.03), systolic BP (*p* = 0.005) and 11-dh-TXB2 urinary levels (*p* = 0.0002). Furthermore, AugP in the first model showed a correlation with male sex (*p* < 0.0001), HbA1c (*p* = 0.02) and HDL cholesterol (*p* = 0.03). In the second model, the variables significantly associated with AugP were male sex (*p* < 0.0001), HbA1c (*p* = 0.02), HDL cholesterol (*p* = 0.01) and 11-dh-TXB2 urinary levels (*p* < 0.05).

## 4. Discussion

In this study, we measured biomarkers of platelet reactivity and inflammation in subjects with prediabetes. Furthermore, we evaluated the association of these factors with qIMT and arterial stiffness, which are well known as early markers of cardiovascular disease and predictive of cardiovascular events.

We found that 11-dh-TXB2 urinary levels were significantly higher in the prediabetes group compared with the control group, as well as MPV; moreover, we found no differences between these parameters in the prediabetes group compared with the NODM group; these data provide evidence of an in vivo increased platelet activation in subjects with early alterations of glucose homeostasis.

Few studies are available concerning the 11-dh-TXB2 marker in subjects with prediabetes. Previous studies highlighted the association between prediabetes and platelet over-reactivity and resistance to antiplatelet therapy [31]. Santilli et al. found an enhanced thromboxane biosynthesis in subjects with impaired glucose tolerance (IGT), a subset of prediabetic subjects. Moreover, they observed that, among IGT subjects, those experiencing conversion to overt diabetes were characterized by a progressive rise in urinary thromboxane metabolite excretion rate [32]

The increase in 11-dh-TXB2, which is a direct marker of platelet activation, was already described in patients with type 1 and type 2 diabetes mellitus. Zaccardi et al. found that asymptomatic young subjects with type 1 diabetes showed persistently enhanced TXA2-dependent platelet activation and oxidant stress in vivo, and this phenomenon was related to female sex and microvascular and oxidative damages [18]. Al-Sofiani et al. demonstrated that type 2 diabetic patients have higher in vivo platelet activation compared with subjects without diabetes both before and after antiplatelet therapy, and they hypothesized that endothelial dysfunction may play a role in this platelet hyperactivity [33]. In addition, Lopez et al. highlighted that type 2 diabetes is associated not only with an increase in platelet reactivity biomarkers, but also with a poor response to antiaggregant therapy [34].

Platelet hyperactivity was observed in other conditions often associated with diabetes and prediabetes, such as obesity, dyslipidemia and hypertension. Davì et al. reported that urinary 11-dh-TXB2 is significantly increased in otherwise healthy obese women, as compared to non-obese controls [35]. Furthermore, the same study group found that successful short-term weight loss was associated with a statistically significant reduction in thromboxane urinary metabolites, identifying insulin resistance as one of the major determinants of platelet reactivity in subjects with obesity [36]. A large body of evidence strongly supports a reciprocal relationship between insulin resistance and endothelial function, sustaining the hypothesis that endothelial dysfunction may be the link between insulin resistance and platelet activation [37]

In addition, previous studies demonstrated that increased total and LDL cholesterol levels are correlated with a higher 11-dh-TXB2 excretion rate, suggesting that platelet activation can be a main determinant of hypercholesterolemia-induced cardiovascular risk [38]. As regards hypertension, Dolegowska et al. highlighted the mutual connections between platelet activation and mean arterial pressure, hypothesizing a potential platelet involvement in atherosclerosis development [39].

Thus, higher urinary thromboxane metabolite excretion is associated with atherosclerosis risk factors and is partially reversible after improved disease control, suggesting that the primary abnormalities, such as hyperglycemia, insulin resistance and hypercholesterolemia, may be responsible for enhanced and persistent thromboxane biosynthesis. In this regard, it is of relevance that disease-modifying strategies, such as lifestyle intervention, different antidiabetic agents and statins, may blunt, at least in part, urinary thromboxane metabolites [38]. Moreover, urinary thromboxane metabolite excretion could be a predictive biomarker of adverse cardiovascular outcomes, as demonstrated in patients with established atherosclerotic cardiovascular disease [40].

The higher MPV in subjects with prediabetes is consistent with other studies such as the one by Inoue et al. that analyzed a Japanese cohort [41]. Furthermore, we found that MPV was directly associated with HbA1c and HOMA-IR, as previously described [42,43]. Insulin resistance is a common denominator of several metabolic conditions related to platelet hyperactivity. This is consistent with the fact that platelets exhibit insulin receptors. Therefore, insulin resistance may cause the lack of the physiological action exerted by insulin on platelet function, such as reduction of the pro-aggregatory properties of agonists, and the activation of endothelial nitric oxide (NO) synthase, with increased NO formation and intraplatelet concentrations of cyclic adenosine monophosphate (cAMP) [36].

As previously reported [44], we highlighted a significant alteration of arterial stiffness and thickness parameters in our prediabetic subjects. Furthermore, we found that 11-dh-TXB2 urinary levels were associated with carotid atherosclerosis and arterial stiffness. Thus, these data suggest a link between platelet over-activation and atherosclerosis in prediabetes, confirming the high cardiovascular risk of this condition. This is clinically relevant, considering the incidence of macrovascular complications is already increased in prediabetes and in new-onset type 2 diabetes [45].

In addition, we found an increase in different inflammatory parameters, such as white blood cell count, hs-CRP, ferritin and fibrinogen. Moreover, even though it did not reach statistical significance, probably because of the sample size, we found an increase in PLR in prediabetic subjects, and we found a significant increase in PLR in diabetic patients compared with controls; furthermore, this parameter was associated with HbA1c and HOMA-IR in the simple regression analysis. This observation is consistent with previous studies in different populations and suggests that prediabetes is a proinflammatory condition [46,47]. Our group previously explored this hypothesis, demonstrating an impaired inflammatory profile in subjects with early glucose intolerance status and metabolic syndrome [6,48]. PLR can be a key factor for emphasizing the interplay between two major components of atherothrombosis: thrombosis and inflammation. Previous studies demonstrated that higher platelet and lower lymphocyte counts are associated with adverse cardiovascular outcomes. Thus, an elevated PLR may have an additive role in predicting major adverse cardiovascular outcomes [22].

This study presents some strengths and limitations. The major strength of the study is the exclusion of patients undergoing therapies known to affect platelet activation such as statins and antiplatelet drugs. Therefore, our results cannot be affected by drugs. As regards limitations, this was a cross-sectional study, and a longitudinal causal relationship cannot be established between changes in plasma platelet activation biomarkers and arterial stiffness and thickness. In addition, there are some differences in the sex distribution between the study groups.

As regards the evaluation of arterial stiffness, we used PWV. However, another indicator has emerged in recent years. The cardio-ankle vascular index (CAVI), which reflects the stiffness of the arterial tree from the beginning of the aorta to the ankle, is easy to measure and operator-independent. Thus, it could be a promising alternative to PWV. In addition, CAVI has been used in the prediabetic and diabetic populations [49,50].

## 5. Conclusions

In conclusion, subjects with prediabetes exhibit increased platelet reactivity as well as a proinflammatory profile. Furthermore, this condition is associated with early markers of cardiovascular disease independently of classical risk factors.

## Figures and Tables

**Table 1:** Clinical and metabolic characteristics of the study population according to glucose tolerance.

	Controls (*n =* 62)	Prediabetes (*n* = 48)	New-Onset Type 2 Diabetes (NODM) (*n* = 60)
**Age (years)**	51.35 ± 7.84	51.75 ± 7.75	54.7 ± 6.84 *^,#^
**BMI (kg/m^2^)**	27.54 ± 3.95	30.39 ± 6.18	30.25 ± 5.22
**SBP (mmHg)**	118.83 ± 11.91	123.37 ± 14.54	131.17 ± 15.05 *^,#^
**DBP (mmHg)**	74.83 ± 9.61	78.54 ± 9.73 *	81.33 ± 8.53 *
**Total cholesterol (mg/dL)**	225 ± 36.23	233.46 ± 36.32	216.62 ± 23.94
**HDL (mg/dL)**	59.55 ± 17.74	54.83 ± 13.8	58.63 ± 37.95
**TG (mg/dL)**	93 (61–118)	114 (84.5–163.5)	108 (78–132)
**LDL (mg/dL)**	137.00 ± 44.61	153.22 ± 34.44	139.9 ± 24.13
**eGFR(mL/min/1.73 m^2^)**	99.25 ± 15.05	98.52 ± 15.55	103.40 ± 21.48
**Fasting glucose (mg/dL)**	85.58 ± 6.61	98.46 ± 16.99 *	127.30 ± 39.33 *^,#^
**Plasma insulin (mIU/L)**	7.10 (5.70–9.30)	9.20 (5.80–11.50) *	10.40 (6.50–19.70) *
**HOMA-IR**	1.46 (1.15–1.95)	2.11 (1.54–3.1) *	3.2 (1.77–5.45) *^,#^
**HbA1c (%)**	5.36 ± 0.23	5.87 ± 0.38 *	6.90 ± 1.19 *^,#^
**ACR (mg/g creatinine)**	7 (6–8)	8 (5–10)	10 (8–18) *^,#^
**Hypertension (%)**	23%	28%	45% *^,#^
**Use of ACEis or ARBs (%)**	13%	25% *	33% *
**Use of CCBs (%)**	3%	6%	13% *
**Use of other antihypertensive drugs (%)**	0%	4%*	3% *
**Active smokers (%)**	20%	36%	31%
**Sex (M/F)**	26/36	30/18 *	42/18 *

The data are presented as the mean ± SD or median (IQR). BMI: body mass index; SBP: systolic blood pressure; DBP: diastolic blood pressure; TG: triglycerides; eGFR: estimated glomerular filtration rate; HOMA-IR: homeostasis model assessment insulin resistance; ACR: albuminuria-to-creatininuria ratio; ACEis: angiotensin-converting enzyme inhibitors; ARBs: angiotensin receptor blockers; CCBs: calcium channel blockers. * *p* < 0.05 vs. controls; ^#^ *p* < 0.05 vs. prediabetes.

**Table 2:** Inflammation and platelet activation indexes in the study population according to glucose tolerance.

	Controls (*n =* 62)	Prediabetes (*n* = 48)	New-Onset Type 2 Diabetes (NODM) (*n* = 60)
**hs-CRP (mg/dL)**	0.09 (0.07–0.29)	0.21 (0.12–0.54) *	0.28 (0.19–0.59) *
**WBC (10^6^/µL)**	6.1 ± 1.8	7.2 ± 1.8 *	7.5 ± 1.8 *
**Ferritin (ng/mL)**	68 (25–125)	119 (73–200) *	108 (40.5–192.5) *
**Fibrinogen (mg/dL)**	324 ± 23.1	350 ± 71.3 *	348.9 ± 42.8
**Platelet count (10^3^/µL)**	230 ± 64.2	251.5 ± 69	255.2 ± 85.4 *^,#^
**MPV (fL)**	8.7 ± 1.1	9.1 ± 0.8 *	9.1 ± 0.8 *
**PLR**	121.2 ± 38	138 ± 54	144 ± 87 *
**11-dh-TXB2 (ng/mg creatine)**	10.6 (6.3–27.6)	16.5(9.9–27.8) *	18.2(8.5;36.6) *^,#^
**8-iso-PGF_2α_ (ng/mg creatine)**	9.7 (6.4–18.3)	9.9 (5.6–13.7)	10.7(6.8–18.2)

The data are presented as the mean ± SD or median (IQR). Hs-CRP: high-sensitivity C-reactive protein; WBC: white blood cells; MPV: mean platelet volume; PLR: platelet-to-lymphocyte ratio; 11-dh-TXB2: 11-dehydro-thromboxane B2 urinary levels; 8-Iso-PGF_2α_: isoprostane PGF_2α_ urinary levels. * *p* < 0.05 vs. controls; ^#^ *p* < 0.05 vs. prediabetes.

**Table 3:** Arterial stiffness and thickness parameters according to glucose tolerance.

	Controls (*n =* 62)	Prediabetes (*n* = 48)	New-Onset Type 2 Diabetes (NODM) (*n* = 60)
**qIMT (mm)**	0.69 ± 0.11	0.75 ± 0.12 *	0.76 ± 0.12 *
**PWV (m/sec)**	7.4 ± 1	7.9 ± 1.5 *	8.1 ± 1.9 *^,#^
**AugP (mmHg)**	10 ± 4.8	12 ± 8 *	12.7 ± 5.5 *
**AugI (%)**	26 ± 8.2	28.8 ± 13.1	30.5 ± 9.7 *
**SEVR (%)**	159.8 ± 27.7	160 ± 25.5	150.4 ± 25.5 *^,#^
**Atherosclerotic plaque presence (%)**	19%	27%	28%

The data are presented as the mean ± SD. qIMT: quality intima–media thickness; PWV: pulsed wave velocity; AugP: augmentation pressure; AugI: augmentation index; SEVR: subendocardial viability ratio. * *p* < 0.05 vs. controls; ^#^ *p* < 0.05 vs. prediabetes.

**Table 4:** Multiple regression analysis evaluating qIMT, AugP and PWV as dependent variables.

	Coefficient β	*p* Value
**qIMT**		
Multiple regression—Model 1 *		
Age (years)	8.9	<0.0001
FG (mg/dl)	1.48	0.01
Multiple regression—Model 2 **		
Age (years)	0.007	<0.0001
11-dh-TXB2 (ng/mg creatinine)	0.002	<0.05
Fibrinogen (mg/dL)	0.0003	<0.05
8-iso-PGF_2α_ (ng/mg creatinine)	−0.003	0.04
**AugP**		
Multiple regression—Model 1 *		
Male sex	−5.97	<0.0001
HbA_1c_ (%)	1.65	0.02
HDL (mg/dL)	−0.04	0.03
Multiple regression—Model 2 **		
Male sex	−7.74	<0.0001
HbA_1c_ (%)	2.46	0.02
HDL (mg/dL)	−0.05	0.01
11-dh-TXB2 (ng/mg creatinine)	0.107	<0.05
**PWV**		
Multiple regression—Model 1 *		
Male sex	0.56	0.03
SBP (mmHg)	0.04	<0.0001
HDL (mg/dL)	−0.12	0.01
Multiple regression—Model 2 **		
Male sex	0.88	0.03
SBP (mmHg)	0.03	0.005
11-dh-TXB2 (ng/mg creatinine)	0.031	0.0002

* Model 1 was adjusted for age, sex, BMI, systolic BP, HDL cholesterol, LDL cholesterol, HbA_1c_, fasting glycemia and HOMA-IR. ** Model 2 was adjusted for 11-dh-TXB2 urinary levels, fibrinogen, MPV, platelet count, hs-CRP, 8-iso-PGF_2α_urinary levels and PLR. FG: fasting glycemia; 11-dh-TXB2: 11-dehydro-thromboxane B2 urinary levels; 8-iso-PGF_2α_: isoprostane PGF_2α_ urinary levels; BMI: body mass index; PLR: platelet-to-lymphocyte ratio; hs-CRP: high-sensitivity C-reactive protein; SBP: systolic blood pressure.

## Data Availability

Data for this study are available under request.
